# Green Chemistry to Modify Functional Properties of Crambe Protein Isolate-Based Thermally Formed Films

**DOI:** 10.1021/acsomega.3c00113

**Published:** 2023-05-31

**Authors:** William R. Newson, Antonio J. Capezza, Ramune Kuktaite, Mikael S. Hedenqvist, Eva Johansson

**Affiliations:** † Department of Plant Breeding, Swedish University of Agricultural Sciences, P.O. Box 190, SE-234 22 Lomma, Sweden; ‡ Department of Fibre and Polymer Technology, Royal Institute of Technology, SE-10044 Stockholm, Sweden

## Abstract

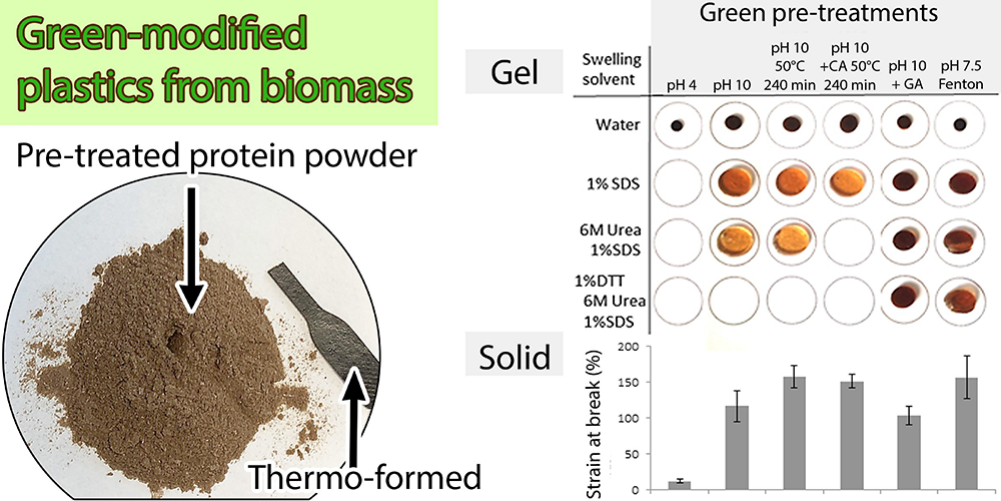

Proteins are promising precursors to be used in production of sustainable materials with properties resembling plastics, although protein modification or functionalization is often required to obtain suitable product characteristics. Here, effects of protein modification were evaluated by crosslinking behavior using high-performance liquid chromatography (HPLC), secondary structure using infrared spectroscopy (IR), liquid imbibition and uptake, and tensile properties of six crambe protein isolates modified in solution before thermal pressing. The results showed that a basic pH (10), especially when combined with the commonly used, although moderately toxic, crosslinking agent glutaraldehyde (GA), resulted in a decrease in crosslinking in unpressed samples, as compared to acidic pH (4) samples. After pressing, a more crosslinked protein matrix with an increase in β-sheets was obtained in basic samples compared to acidic samples, mainly due to the formation of disulfide bonds, which led to an increase in tensile strength, and liquid uptake with less material resolved. A treatment of pH 10 + GA, combined either with a heat or citric acid treatment, did not increase crosslinking or improve the properties in pressed samples, as compared to pH 4 samples. Fenton treatment at pH 7.5 resulted in a similar amount of crosslinking as the pH 10 + GA treatment, although with a higher degree of peptide/irreversible bonds. The strong bond formation resulted in lack of opportunities to disintegrate the protein network by all extraction solutions tested (even for 6 M urea + 1% sodium dodecyl sulfate + 1% dithiothreitol). Thus, the highest crosslinking and best properties of the material produced from crambe protein isolates were obtained by pH 10 + GA and pH 7.5 + Fenton, where Fenton is a greener and more sustainable solution than GA. Therefore, chemical modification of crambe protein isolates is effecting both sustainability and crosslinking behavior, which might have an effect on product suitability.

## 1 Introduction

Increasing awareness about sustainability issues has raised the interest for bio-based and “green” alternatives to replace petroleum-based plastics. Proteins (e.g., from feathers, bone meal, wheat gluten, cottonseed, and soy) are especially interesting biomacromolecules in this context due to their abundance as process side streams (e.g., cottonseed meal from cotton oil production) and because of their variety of available chemical and structural elements.^1^ Common industrial processing methods, i.e., dispersion coating, extrusion, injection, and compression molding, have been found suitable for the production of protein-based plastics, thereby allowing large-scale production.^2−8^ Several possible applications have been indicated for the protein-based plastics, including agricultural products like mulch films,^9^ planting pots,^10^ food packaging films,^11^ and superabsorbents,^12^ and they can be useful even for medical biomaterials, e.g., as cellular scaffolds.^13^

The world oilseed production reached 601 million tonnes in 2020/21, while after oil extraction, the worldwide oilseed meal production reached 351 million tonnes.^14^ Currently, the main use for the oilseed meal is as animal feed. Novel types of crops, e.g., crambe (*Crambe abyssinica* Hochst), are evaluated for their potential as oilseed crops due to an increasing demand for high-value industrial oil and because of the requirements of petroleum replacers. High content of anti-nutritional compounds in these industrial oilseeds makes their meal non-suitable for human and animal consumption. Therefore, the development of non-feed industrial applications is a prerequisite to improve the economic viability of these residual meals to contribute to the bioeconomy.^15^ Thus, novel protein-based materials are of emerging importance. However, protein-based materials developed directly from the crambe meal have until now showed poor performance,^8^ although blends with wheat gluten have shown some promise.^4,16^ The crambe seed shares the main storage protein groups of other brassicas, i.e., their proteins consist of cruciferin (12S globulin) and napin (2S albumin).^17,18^ Previous studies on thermal processing of proteins from crambe have shown that cruciferin-rich fractions have a substantial ability to aggregate on heating, while the napin fraction is resistant to aggregation at a process temperature of 130 °C.^15^

Despite the multiple reports on the beneficial properties of protein-based materials, several issues have been reported that need to be overcome before they can be acceptable plastic substitutes. Such issues are, e.g., lack of purity, hydrophilicity, complex chemical and structural make-up, and sensitivity to environmental conditions. Various processing conditions and modifications have been tested to improve the performance of protein-based materials from brassicas, such as protein isolation,^15^ plasticization,^8^ denaturation (thermal and chemical),^19^ chemical reduction,^8^ crosslinking,^1^ and side group addition.^12,20^ In terms of chemical crosslinking of various protein-rich sources, aldehydes, which predominantly act on lysine,^21^ have been a common choice as a crosslinking agent despite concerns about their toxicity^22^ and lack of control over their specific mechanism.^23^ Alternative “greener” crosslinking schemes have been proposed for various proteins, applying multifunctional carboxylic acids, e.g., citric acid, which also acts on lysine.^24^ Another green crosslinking route has been proposed by forming dityrosine by oxygen radicals formed with the photo-Fenton reaction.^25,26^ Through the isolation of proteins in solution, opportunities increase to modify proteins so that reaction sites become more accessible, allowing crosslinking before drying the product for thermal processing in the solid state.^1^ Through such processes, a modified protein isolate could be made available, suitable for industrial techniques.

Previous studies on crambe protein have shown that a combination of alkali extraction and isoelectric precipitation to produce protein concentrates has a potential for production of molded films.^15^ However, additional modification or functionalization of the proteins are needed to further improve the properties of the produced materials. Thus, the purpose of the present study was to increase the understanding on opportunities to modify the performance of the crambe proteins by chemical modification of the proteins before thermal processing. Therefore, we examined the effect on protein size distribution, crosslinking, structural features, swelling, and tensile performance of treatments, including crosslinking agents, applied to the crambe protein in solution followed by thermal processing. Furthermore, relationships between protein crosslinking, swelling behavior, and tensile performance were evaluated. The multitude of treatments evaluated in the present study and the wide array of experimental measurements of protein behavior connected to the variation in tensile properties allowed us to model options for greener solutions in terms of protein-based materials as presented in the present paper.

## 2 Materials and Methods

### 2.1 Materials

Crambe seed, including the pod, was obtained from the Plant Research Institute (PRI, Wageningen, Netherlands). Oil was extracted and the seed was ground following the petroleum distillate method of Appelqvist.^27^ The final meal has a moisture content of 11.1 ± 0.02% (dry basis) and particle size <500 μm. Urea, SDS, citric acid, and trisodium citrate were supplied by Duchefa (Haarlem, Netherlands), monosodium phosphate (NaH_2_PO_4_·H_2_O) by J.T. Baker (Deventer, Netherlands), dithiothreitol (DTT) from Saveen Werner (Limhamn, Sweden), and glycerol (99.5% purity) by Karlshamn Tefac AB (Karlshamn, Sweden). Glutaraldehyde (25% solution), hydrogen peroxide (30% solution), and trifluoroacetic acid (TFA, spectroscopy grade) were supplied by Merck (Darmstadet, Germany), and acetonitrile was obtained from Sigma-Aldrich (Steinheim, Germany). All water was purified by a ThermoScientific GenPure Pro system (ThermoElectric, Langenselold, Germany)*.* The pH was adjusted by adding 1 M and 0.1 M NaOH and HCl as needed.

### 2.2 Protein Isolation

In the present study, protein isolates were produced using a combination of alkali extraction and isoelectric precipitation. The methodology chosen was based on previous results,^15^ evaluating extraction routes to produce and characterize protein concentrates and isolates from crambe as well as the properties of films produced from these concentrates/isolates. Basically, the combination of alkali extraction with isoelectric precipitation resulted in protein isolates with a high total protein content (ca. 90%) consisting of both 2S napin and 12S cruciferin and a high level of protein–protein interaction and high tensile strength in films produced thereof.^15^ Briefly, the protein isolate was produced in several batches through a five-step procedure consisting of acid extraction (with 0.1 M HCl) of the deoiled crambe meal at pH 3 (20:1, solvent vol:meal mass) for 30 min with stirring at room temperature (RT) followed by centrifugation at 12,000 RCF for 30 min at RT, and the supernatant was discarded. The residual solids were extracted at pH 11 for 30 min with stirring at RT followed by centrifugation at 12,000 RCF for 30 min at RT, and the supernatant and residuals were retained. Residuals from initial pH 11 extractions were re-extracted at pH 11 and 10:1 (solvent vol:original meal mass). The supernatants were combined and adjusted to pH 4 under stirring for 30 min and rested without stirring for 30 min followed by centrifugation at 12,000 RCF for 30 min at RT, discarding the supernatant. The resulting pellet was dispersed in water and washed at pH 4 (10:1, solvent vol:original meal mass) by stirring for 30 min followed by centrifugation at 12,000 RCF for 30 min at RT. The isolates were then lyophilized, and all batches were homogenized by grinding (IKA A10, IKA, Germany).

### 2.3 Protein Modification

In the present study, six treatments were used to modify the protein structure, to further evaluate the effect of such modifications on the performance of thermally produced films. In all cases, the start material was a lyophilized protein isolate, which was resuspended in water (7% w/v). The six different treatments were as described below:

1. No modification. The isolate produced at pH 4 was simply resuspended in water (ISO) and then lyophilized again (as described above).
2. Increasing the pH to 10 (pH 10).
3. Increasing the pH to 10 followed by heating to 50 °C for 240 min (pH 10/50).
4. Increasing the pH to 10 and adding 2.5% glutaraldehyde (one of the most commonly used crosslinking agents, although moderately toxic) at RT (pH 10/GA).
5. Increasing the pH to 10 followed by the addition of 5% (g/g protein) citric acid and heating to 50 °C for 240 min (pH 10/50/CA).
6. Adding 5.7% trisodium citrate monohydrate and 2.7% FeSO_4_·7H_2_O, adjusting the pH to 7.5, and adding 1.3% hydrogen peroxide (Fenton reaction, FEN).

All percentages are on a protein isolate basis. After treatment, protein solutions were lyophilized and ground to a fine powder before further treatments.

### 2.4 Crambe Protein-Based Plastic Compression Molding

Compression molding was carried out similar to that of Newson et al.^8^ The treated and lyophilized proteins were blended with 30% glycerol by hand in a mortar and pestle, approximately for 2 min. The plasticized protein was placed between preheated aluminum plates using polyethylene terephthalate release sheets and an aluminum frame with a 10 cm × 10 cm opening and a thickness of 0.5 mm to control the resulting film size. The assembly was pressed for 5 min at 130 °C and a force of 100 kN. Specimen pH 10/50 was pressed in a 7.5 cm × 7.5 cm × 0.5 mm frame at 56 kN. After pressing, the film was immediately placed between RT aluminum plates for cooling.

### 2.5 Molecular Weight Distribution (SE-HPLC)

Size exclusion high-performance liquid chromatography (SE-HPLC) was carried out as previously reported by Newson et al.^19^ Briefly, samples were cut by hand into pieces not larger than 250 μm, and 16.5 mg was placed in a 1.5 mL Eppendorf tube with 1.4 mL of extraction buffer, 0.5% SDS, and 0.05 mol NaH_2_PO_4_ at pH 6.9. The samples were serially extracted using three steps: (1) 10 s of vortexing and 5 min of shaking (2000 rpm, IKA VXR Basic, IKA Werke, Germany), (2) 30 s of sonication, and (3) 30 + 60 s of sonication (both sonications at an amplitude of 5 μm) (Sanyo Soniprep, Tamro, Sweden). Extraction steps 2 and 3 were preceded by manually breaking up the pellet. Each extraction was followed by centrifugation at 16,000 RCF for 30 min at room temperature and decanting of the supernatant directly into HPLC vials. All extractions were performed in triplicate.

**Figure 1:**
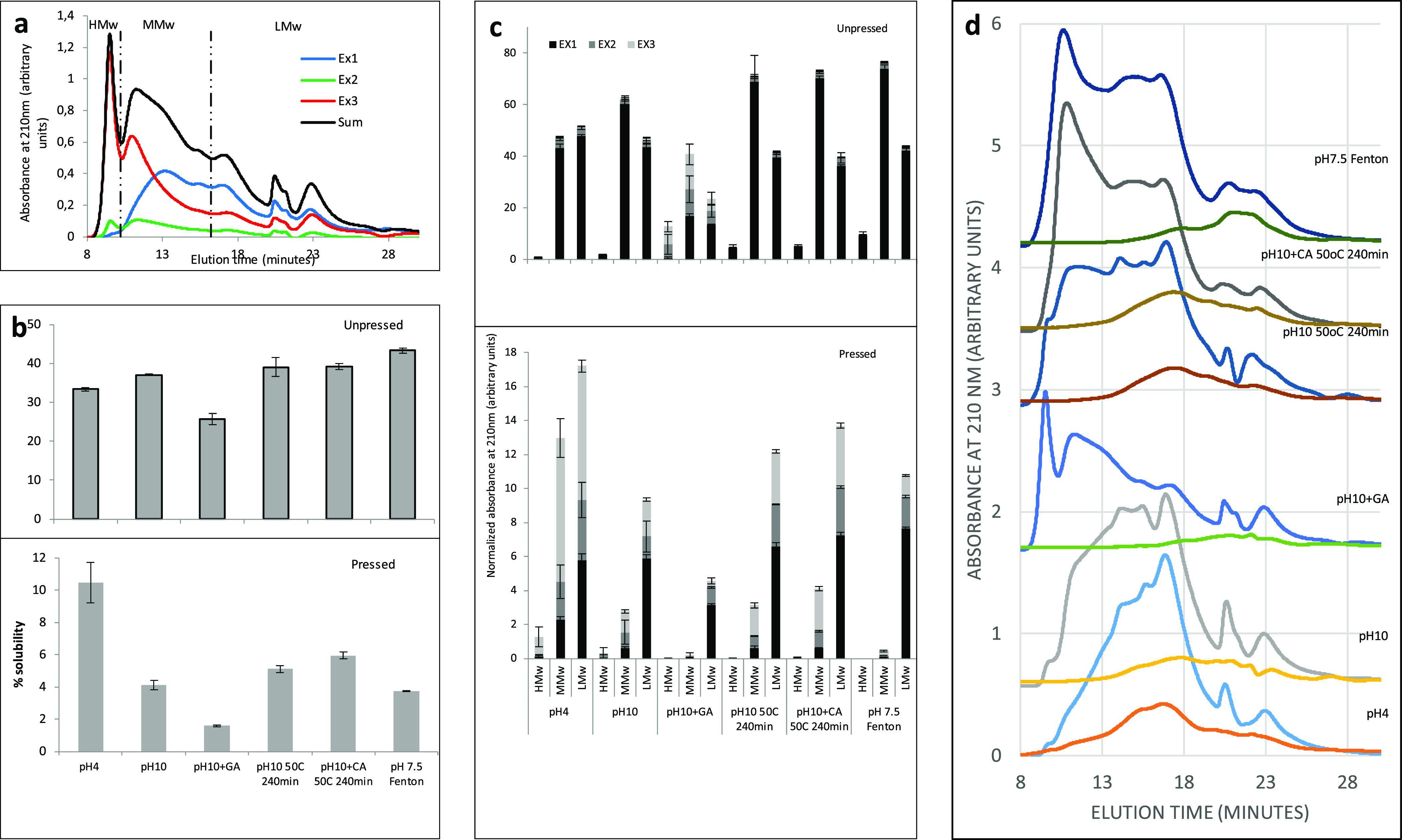
Results from SE-HPLC analyses showing (a) example chromatograms of unpressed pH 10 + GA sample from three steps of extraction and a summary of these extractions depicting HMw (8–12.2 min), MMw (12.2–16.2 min), and LMw (16.2 to 30 min) proteins, (b) total relative (as compared to protein extracted from crambe flour) extractable protein from pressed and unpressed samples, (c) normalized (to total extractable protein of pH 4 unpressed sample) HMw, MMw, and LMw proteins over three extractions in unpressed and pressed samples, and (d) chromatograms for the sum of all three extractions. Darker curves indicate unpressed samples, and lighter curves indicate pressed samples.

Separations were carried out on a Waters 2695 control unit and 996 photodiode array detector using a prefilter (SecurityGuard GFC 4000, Phenomenex, USA) and Biosep-SEC-S4000 column (Phenomenex, USA) at 20 °C. Data were 3D blank-corrected using the extraction buffer and chromatograms extracted at 210 nm (Empower v2, Waters, USA). Chromatograms were divided into three domains: high molecular weight (HMw) from 7.5 to 10.2 min, medium molecular weight (MMw) from 10.2 to 16.2 min, and low molecular weight (LMw) from 16.2 to 30 min (see Figure 1a).

### 2.6 Structure Determination

Infrared spectra were recorded averaged over 16 scans using a Spectrum 2000 FTIR spectrometer (PerkinElmer, USA) in attenuated total reflection, single reflection mode (Golden Gate, Specac, UK). Samples were dried for at least 72 h over silica gel before testing. The spectra were recorded from 4000 to 600 cm^–1^. Data were Fourier self-deconvoluted (Spectrum ver. 3.02, PerkinElmer, USA) using a γ of 2 and a smoothing factor of 70%. The deconvolution/curve resolution of the amide I region (1700–1580 cm^–1^) was performed as reported by Cho et al.^6^ The deconvoluted data were treated using Origin 9.1 (Microcal Software Inc.) for each sample. The peak resolution consisted of fitting nine Gaussian peaks, which were initially centered and fixed at 1618, 1625, 1634, 1644, 1651, 1658, 1667, 1681, and 1693 cm^–1^. An additional peak at 1595 cm^–1^ (−NH_2_ scissoring) was considered in the fitting. However, the area of the peak was always <1% and was therefore not considered for the calculations. The peak centers were unfixed selectively and allowed to move ±1 cm^–1^ when the *r*^2^ of the total fitted curve was below 0.999. The iteration was stopped when an *r*^2^ > 0.9994 was obtained. The secondary structure content was calculated as the relative area (%) of the resulting Gaussian peaks.

### 2.7 Swelling

Disks of 3 mm in diameter were punched from pressed films of treated crambe isolate. Disks were stored over freshly prepared silica gel to constant weight before immersion. Disks were immersed in aqueous solutions (5 mg/mL) in triplicates for 60 days at 22 °C. Solutions used were as follows: water, 1% SDS, 1% SDS + 6 M urea, 1% SDS + 6 M urea + 1% DTT, all with 0.02% sodium azide to prevent microbial growth. The four solutions selected for swelling of thermally processed modified protein films, with each used to disrupt a specific type of interaction holding the protein network together, were as follows: water for hydrogen bonding, SDS for disrupting charge interactions, urea + SDS to denature the secondary structure, and DTT + urea + SDS to reduce disulfide bonds. Disks were removed from the solution, surface water was removed with dry filter paper (Munktell #3, Ahlstrom-Munksjo, Sweden), and the disks were weighed.

### 2.8 Tensile Testing

Tensile testing was carried out as previously described by Newson et al.^19^ Briefly, tensile specimens were punched from crambe isolate films (using the standardized method ISO 37-type 3, Elastocon, Sweden), resulting in dumbbell-shaped specimens with a total length of 64 mm and a narrow section, which is 16 mm long and 4 mm wide.^4^ These specimens were then conditioned at 23 °C and 50% relative humidity for 48 h. Specimens were tested on an Instron 5566 test machine with a 500 N load cell, using Bluehill software (Instron AB, Sweden), at 10 mm/min under the conditioning environment. All values were calculated from a minimum of seven replicates.

## 3 Results and Discussion

A summary of mean values with standard deviation for all measured properties of the unpressed and pressed samples is given in Table S1.

### 3.1 Protein Molecular Weight Distribution in Unpressed Samples

The protein molecular weight distribution increased by each extraction step (Figure 1a shows extractions from unpressed pH 10-GA isolate as one example) for all unpressed samples evaluated in this study, as has also been reported in earlier studies on various plant-based materials.^15,28,29^ The higher molecular weight distribution and the need for more severe conditions to extract the proteins are both verifications of increased crosslinking of the proteins extracted.^28,30^

### 3.2 Modification of Protein Crosslinking by pH

The effect of utilizing pH as a tool to modify the proteins was clearly seen as a change in the molecular weight distribution in the unpressed samples (Figure 1b–d). The modification of the proteins by the use of pH 10 instead of pH 4 resulted in an increase in the total amount of extractable proteins (Figure 1b). The reason for the increased amount of proteins extracted was mainly the increased extraction of MMw proteins (Figure 1c), as was also detected as a result in the summed chromatograms (Figure 1d).

**Figure 2:**
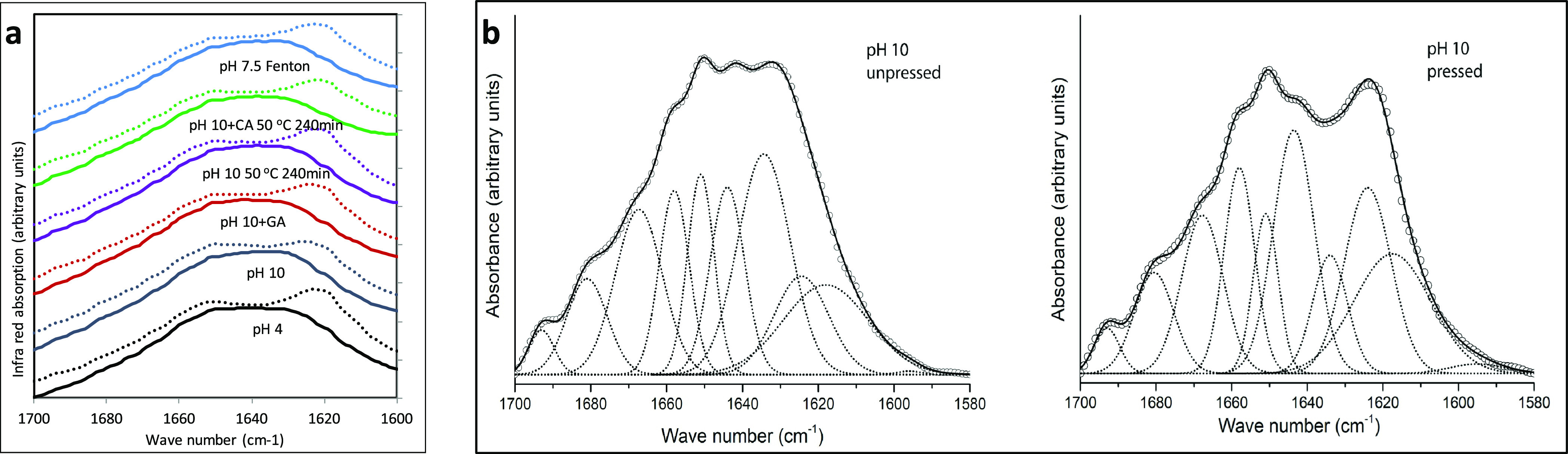
Infrared absorption data for (a) glycerol-plasticized powder (unpressed) and pressed films and (b) unpressed and pressed pH 10 samples with Fourier self-deconvolution, baseline correction, and added fitting Gaussian curves. Legends in panel (a) denote the condition for the production of the modified protein isolate. Solid lines (a) indicate unpressed material, and dotted lines indicate pressed material. Data normalized to the total amide I area. Open circles (b) indicate original data after Fourier self-deconvolution and baseline correction, circles indicate experimental data, the solid line indicates the composite-fitted curve, and dotted lines indicate individually fitted Gaussian curves.

These results corresponded well with secondary structures (Figure 2a) of the unpressed samples, fitting the FT-IR data to Gaussian curves (Figure 2b), resulting in a higher proportion of strongly H-bonded β-sheets obtained for pH 4 samples compared to pH 10 samples (Table 1). Thus, molecular weight distribution data and FT-IR data indicated the higher crosslinking of the proteins in pH 4 unpressed samples than in pH 10 unpressed samples. Previous studies have shown the favor of a low crosslinking degree in unpressed samples, making cysteins available for crosslinking at pressing, resulting in improved tensile properties of films produced.^31−33^

**Table 1:** Results of Curve Fitting Secondary Structures to the Deconvoluted IR Absorption Spectra of the Amide I Band^a^

		relative area of amide I band from the Gaussian component (%) for different treatments											
		pH 4		pH 10		pH 10 + GA		pH 10, 50 °C, 240 min		pH 10 + CA, 50 °C, 240 min		pH 7.5, Fenton	
average position (SD)	assignment	U	P	U	P	U	P	U	P	U	P	U	P
1618 + 1625	β-sheets, strongly H-bonded	29	38	24	33	26	35	24	34	26	34	23	36
1634 + 1680	β-sheets, weakly H-bonded	27	10	28	15	25	11	26	11	20	9	30	7
1644	unordered	10	21	12	18	14	20	12	23	15	21	8	23
1651	α-helices and random coils	9	5	10	7	9	7	10	5	12	6	12	5
1658	α-helices	11	10	10	12	10	8	7	10	9	8	8	6
1667 + 1691	β-turns	14	14	16	14	16	17	21	16	19	20	17	22

a U, unpressed; P, pressed.

Changes to a basic pH at film pressing are also known to modify the charge distribution of the amino acids in the proteins, especially making lysine more reactive.^34,35^ Changes of charges of amino acids might explain the often seen increase in structure (e.g., β-sheets) and tensile properties in films produced at basic pH, as a result of an increase in bond (disulfide and isopeptide) formation.^36−38^ Here, the pressing of the samples resulted in a decrease in protein solubility (Figure 1b) and an increase in strongly H-bonded β-sheets and unordered secondary structures (Table 1) for samples produced at both pH 4 and 10. However, the decrease in solubility was higher (Figure 1b), and the changes in secondary structures included a higher increase in strongly H-bonded β-sheets, a less decrease in weakly H-bonded β-sheets, and a lower increase in unordered structures for pH 10 samples compared to pH 4 samples (Table 1), corresponding well to results in previous studies.^8,19^ Several previous studies have reported a close correlation between changes in protein solubility and protein secondary structures,^33,39^ as also shown in the present study. The decrease in protein solubility was mainly due to a decrease in solubility of larger-sized (HMw and MMw) proteins and later sequential extractions (Figure 1c,d), indicating a higher degree of crosslinking in pressed pH 10 samples than in pH 4 samples, also previously reported.^15,37^

**Figure 3:**
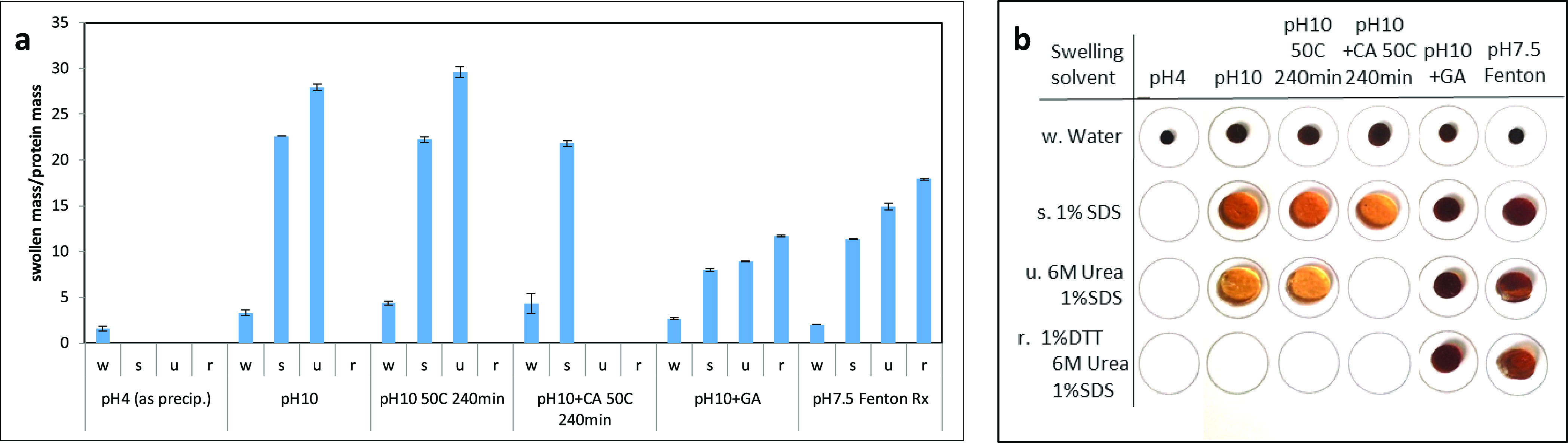
Results on samples swelling on immersion, showing (a) mass gain and (b) swollen disks from pressed films. Empty columns and circles denote samples that dissolved on immersion.

The chemical state of the proteins is well known to affect the liquid uptake capacity of the proteins and, therefore, their swelling behavior.^40−42^ A crosslinked macromolecular network exposed to a solvent is known to swell until equilibrium is reached between osmotic forces favoring the absorption of more solvent and the resistance of the network to further expansion.^43^ In the present study, the pH 10 samples showed higher absorbing capacity than the pH 4 samples of water, 1% SDS solution, and 6 M urea + 1% SDS solution (Figure 3a). However, the water treatment resulted basically in a minor equilibrium swelling (Figure 3b) for samples at both pH values, indicating the hydrophobic character of the proteins also previously reported,^44^ although a crosslinked network may also hinder swelling by being extensive. Surfactants such as SDS are known to unfold proteins through binding to hydrophobic and positively charged residues of the proteins.^45^ In this study, SDS treatment of the pH 4 samples led to loss of cohesion of the proteins, indicating a weak network connected by charged residues, while pH 10 samples were able to swell and maintained integrity.

**Figure 4:**
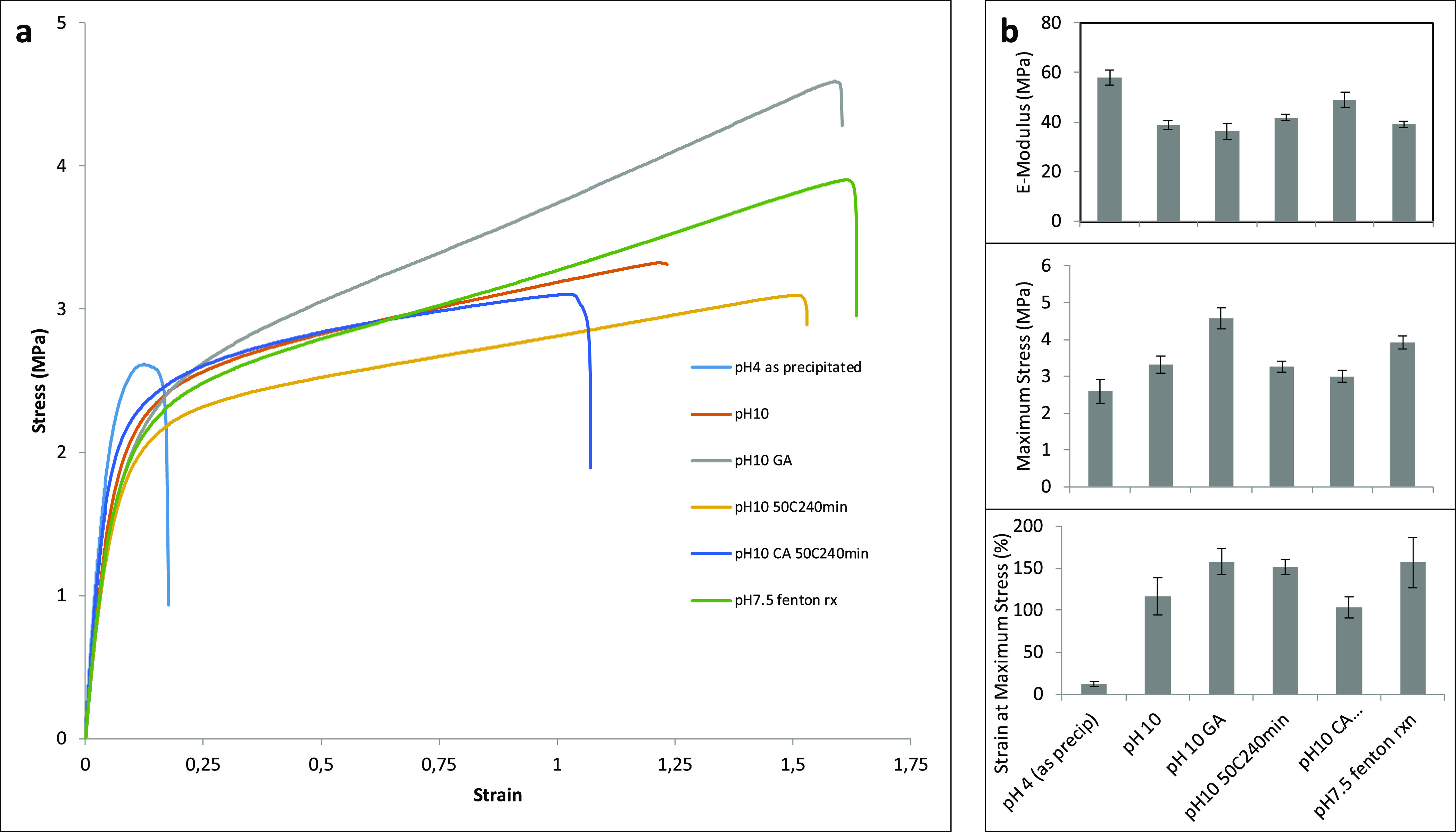
Mechanical properties presented as (a) stress–strain curves and (b) mean values of E-modulus, maximum stress, and extensibility of protein films from modified samples.

Additional swelling was obtained in the pH 10 samples while being treated with the chaotropic agent urea. Urea is known to disrupt the secondary structure by interfering with hydrogen bonding and hydrophobic interactions, thereby allowing network extension.^46,47^ In other studies, basic conditions have also been found favorable for production of protein-based superabsorbent materials with high uptake of water, saline solutions, and blood.^35,41,42^ Similarly, as reported in previous studies,^19,48^ the samples produced under basic conditions (pH 10) resulted in a more crosslinked network with higher tensile strength, here shown by increased strain at maximum stress and maximum stress values as compared to pH 4 samples (Figure 4a,b).

### 3.3 Modification of Protein Crosslinking by the Crosslinking Agent GA

Adding GA to the crambe protein in pH 10 solution resulted in an overall decrease in protein solubility in unpressed samples compared to crambe protein samples in pH 10 without the addition of GA (Figure 1b). The decrease in protein solubility was mainly due to the decreased extractability of medium- and small (MMw and LMw)-sized proteins (Figure 1c). However, the extractability of the HMw proteins was actually increased in unpressed GA samples as compared to samples without GA in pH 10 solution (Figure 1c). The overall decrease in protein extractability shifts toward a higher extractability of large proteins, and a decrease in the solubility of medium and small proteins in unpressed GA samples is clearly seen when visualized in Figure 1d and is an indication of the proteins being increasingly crosslinked. Furthermore, the GA treatment contributed to proteins being more difficult to extract in unpressed samples, thereby responding to sonication to a larger extent than the other samples evaluated in the present study (Figure 1c), indicating a crosslinked network. Although the crosslinking process may differ, GA is known as a strong crosslinker, since GA is shown to hold 13 different forms in solution, thereby reacting with proteins in different ways.^49^ Secondary structure analysis by FT-IR indicated a slight increase in strongly H-bonded β-sheets and a slight decrease in weekly H-bonded β-sheets with GA treatment in unpressed samples, further indicating the crosslinking as a result of the GA treatment (Table 1).

Similar to pH 4 and pH 10 samples, pressing of the pH 10 + GA samples resulted in a decrease in protein solubility (Figure 1b) and an increase in strongly H-bonded β-sheets and unordered secondary structures (Table 1). However, the change in secondary structure was similar to the pH 10 samples also when GA was added. Despite the lack of change in secondary structure by adding GA, clear differences in molecular weight distribution were noted. Pressing of the GA samples resulted in the lowest amount of soluble proteins among the samples (Figure 1b), with basically no HMw and MMw proteins and low levels of SMw proteins extracted (Figure 1c), indicating pressed GA samples to be the most crosslinked samples in this study. Thus, GA contributed not only to crosslinking in unpressed samples but also to the most developed crosslinking while pressing. Previous studies have depicted GA as an important crosslinker that contributes to improved reinforcement in a range of proteins and composite films.^50−53^ At the pressing of samples, GA is known to act as a crosslinker on lysine at elevated pH but also on tyrosine and arginine.^21,54−56^ The pressed pH 10 + GA samples showed high resistance to dissolution in various solvents, although with relatively limited swelling compared to the rest of the samples evaluated here (Figure 3). Again, this indicates a strong protein network in the samples. In previous studies, swelling in relation to dissolution has clearly been shown as an indication of the protein network formation.^35,57^ This fact was further elucidated by an SE-HPLC analyses of the swelling liquid at equilibrium, showing less protein in the pH 10 + GA sample than in any of the other samples. Due to the high protein crosslinking in the pH 10 + GA samples, tensile properties were generally favorable as compared to the other samples evaluated with high values both for maximum stress and strain at maximum stress (Figure 4). The correspondence of protein crosslinking with tensile strength has been shown in a number of publications on other protein-based materials and using a range of additives.^7,36,37^

### 3.4 Modification of Protein Crosslinking by Heat Incubation

In previous studies, pressing samples at high temperature and basic pH conditions has resulted in increased polymerization of plant proteins by the formation of disulfide bonds through oxidation or SH-SS interchanges.^48,58,59^ In this study, unpressed samples were treated with a combination of pH 10 and 240 min incubation at 50 °C, which did not have a large effect on the solubility of the proteins as compared to the pH 10 treatment without heat incubation (Figure 1b), indicating no changes in disulfide bond formation. With pressing, heat-treated samples at pH 10 resulted in higher protein solubility and exchange toward lower molecular weight distribution in proteins extracted than non-heat-treated samples (Figure 1b,c). Thus, a moderate heat treatment at basic pH did not contribute to an increase in crosslinking through disulfide bond formation for the crambe protein samples in the present study. The mechanisms behind heat treatment-induced crosslinking are mainly related to the unwinding of secondary structures of the proteins and breaking of present crosslinks so that novel and additional crosslinks are able to form.^60^ The temperature needed to have an impact on crosslinking reactions differs for various proteins, as do the ability of the proteins to crosslink. For the crambe proteins, secondary structure evaluations by FT-IR did not reveal any clear signs of increased crosslinking from moderate heat incubation at pH 10 before pressing. However, heat incubation led to an increased number of β-turns and unordered structures at the cost of weakly bonded β-sheets and α-helices in unpressed and pressed samples, respectively (Table 1 and Figure 2). Also, the swelling of the heat-incubated samples at pH 10 resembled to a high extent the swelling of the non-heated pH 10 samples (Figure 3a,b), indicating no extended crosslinking in the heat-treated samples. However, heat incubation led to higher strain at maximum stress, as compared to non-heat-induced pH 10 samples (Figure 4a,b), indicating a more extensible network with heat incubation reflecting the increase in unordered structures. This again indicated that the moderate heat induction at pH 10 before pressing did not lead to crosslinks for crambe protein samples, as previously shown for other protein sources treated with high temperatures at basic pH during pressing.^48,58,59^ However, also, a moderate temperature treatment at basic pH was found to contribute some changes in protein structures for the crambe proteins.

### 3.5 Modification of Protein Crosslinking by Heat Incubation Combined with Citric Acid (CA)

The addition of CA for modifications of the proteins at pH 10 and heating at 50 °C for 240 min did not change the total extractability of the proteins (Figure 1b) nor the content of the different fractions (Figure 1c) compared to if CA was not added. As CA is a weak acid (with a pH of around 3.5), the addition of 5% CA to a sample with a pH of 10 will not significantly reduce the pH of the sample, which might be the reason for lack of changes in the extractability of the proteins. However, previous studies have revealed increased Mw in wheat protein in solution by CA addition^24^ due to ester linkage formation between CA and lysine. Also, the summed chromatograms (Figure 1d) visualize a shift to higher Mw within the HMw interval, indicating an increase in crosslinks of the HMw proteins with the addition of CA. The FT-IR results verified these increases in crosslinks, showing an increase in strongly H-bonded β-sheets and unordered structures at the cost of weakly H-bonded β-sheets in CA samples compared to similar samples (pH 10 and heating at 50 °C for 240 min) without CA (Table 1).

For pressed samples, the addition of CA resulted in the increased solubility of the MMw and LMw proteins compared to similar samples (pH 10 and heating at 50 °C for 240 min) without CA (Figure 1b,c). Secondary structure results from FT-IR revealed a clear increase in β-turns in these samples compared to similar samples (pH 10 and heating at 50 °C for 240 min) without CA (Table 1). Thus, the pressing of the samples seemed to reduce the crosslinking of the medium- and smaller-sized proteins in CA samples compared to non-CA samples, indicating differences in the crosslinking of these proteins in samples with and without CA.

Urea as a chaotropic agent disrupted and disintegrated the CA film (Figure 3a,b). As a chaotropic agent, urea should disrupt the secondary structure by interfering with hydrogen bonding and hydrophobic interactions, thereby unfolding the protein, allowing further network extension and thus increased liquid uptake.^61^ However, in previous work, urea has also been used as a strong agent together with DTT and heat to break any possible disulfide bond, although irreversible sulfur (S−) or peptide bonds are highly resistant to such treatments.^32^ In the present study, we expected ester bonds to be formed by adding CA to be resistant to urea. However, the treatment with CA and, thereafter, the tough treatment of pressing had a negative impact on the protein crosslinking, which obviously also negatively impacted the liquid uptake (Figure 3) and the tensile performance (Figure 4). Both tensile strength and strain at maximum stress were reduced in films with the CA addition compared with similar films but without CA (Figure 4). Thus, the present protein matrix did not seem to support the formation of ester bonds by adding CA.

### 3.6 Modification of Protein Crosslinking by the Application of the Fenton Reaction

The addition of citrate/iron sulfate/peroxide (Cit/Fe/Pox = Fenton) to the unpressed samples at a pH of 7.5 resulted in the high solubility of proteins at all molecular weights (Figure 1c) and, therefore, in the highest total protein solubility among samples (Figure 1b) as also visualized in Figure 1d. However, after pressing, the protein solubility decreased dramatically in the Fenton samples (Figure 1b) and, in principle, only LMw proteins were extractable (Figure 1c), indicating crosslinking taking place. The Fenton system produces superoxide radicals through the Fenton reaction,^62^ resulting in tyrosine crosslinking.^63^ Another possible effect of Fenton is physical crosslinking through Fe^2+^ ion coordination bonds.^64^ Secondary structure analyses of the proteins (FT-IR) revealed minor differences between the unpressed Fenton and pH 10 samples. However, after pressing, the Fenton samples showed a low degree of α-helices and weakly H-bonded β-sheets, and instead, they were rich in strongly H-bonded β-sheets, β-turns, and unordered structures (Table 1). This, together with the HPLC data (Figure 1), indicates the formation of rather strong character crosslinks. The Fenton samples were also the only samples that showed absorbent properties when any of the four liquids were tested, although with somewhat less expansion than some of the other samples (Figure 3). Not even the 1% DTT + 6 M urea + 1% SDS solution disintegrated and disrupted the samples, indicating the presence of strong crosslinks like peptide bonds or other irreversible bonds.^32,33,65^ This corresponds well with previous studies showing that oxidizing systems such as Fenton or XYZ hypochlorite are known to contribute to the crosslinking of the proteins.^66^ In general, the present study indicates that the pH 10 + GA samples and Fenton samples showed the highest degree of crosslinking, although the type of crosslinking differed with primarily covalent bonds in the pH 10 + GA samples and irreversible/peptide bonds in the Fenton samples. In this study, a pH of 7.5 was applied for the Fenton-treated samples due to a need to keep Fe in solution in its Fe^2+^ state.^66^ In a previous study, a decrease in pH has been shown to result in the lower reactivity of proteins.^67^ However, in the present study, the decrease in pH from 10 to 7.5 in the Fenton samples did neither decrease the protein solubility nor shift the Mw distribution, indicating that the reactivity of the proteins was not negatively influenced. Tensile tests on the Fenton samples resulted in high tensile strength and strain at maximum stress, verifying a high degree of crosslinking in the material.

## 4 Conclusions

Crambe, with a high-quality oil, is a potentially useful crop for the oil industry, leaving a protein-rich residue behind with limited use for food and feed due to the high level of anti-nutritional components. Opportunities to use this protein-rich residue for materials production have until now been limited due to lack of performance of the materials. To overcome these limitations, proteins need to be isolated and the crosslinking behavior of the proteins needs to be improved to increase the functional properties of the proteins. Here, we are for the first time evaluating opportunities to use chemical modifications on the isolated crambe protein to improve the crosslinking behavior and functional properties of the protein. The level of crosslinking between crambe proteins is improved after treatments in solution, applying (i) pH 10 + GA or (ii) pH 7.5 + Fenton, especially as compared to only pH treatments of the proteins in solution. However, the mentioned two treatments result in different types of crosslinking, where pH 10 + GA samples contain a high level of covalent bonds, while pH 7.5 + Fenton samples contain a higher degree of peptide/irreversible bonds. GA is known as a strong crosslinker, although moderately toxic, with more than 13 forms in solution, although for crambe proteins, GA may crosslink on lysine at elevated pH and on tyrosine and arginine, which might explain the heavily crosslinked properties of the proteins after treatments. Due to the highly crosslinked structure, GA-treated samples at basic pH show high tensile strength and good swelling properties in various liquids including even reducing agents. Fenton contributes with tyrosine crosslinking and/or physical crosslinking through Fe^2+^ ion coordination bonds to the proteins, which results in a strong protein network that does not even disintegrate by reducing agents such as the commonly used DTT. Additional treatments to pH 10 + GA of the crambe proteins in solution, e.g., heating or citric acid treatment, did not contribute crosslinking to the level obtained for pH 10 + GA and pH 7.5 + Fenton samples. Thus, the additional treatments created no additional disulfide or ester bonds. The fact that pH 10 + GA and pH 7.5 + Fenton were the two treatments resulting in the best performance, although based on different chemical reactions, calls on additional studies to unlock the chemistry behind these results. Crosslinking density might be one among other reasons such as the 3D structure of the proteins and locations of crosslinks and number of protein subunits involved in the network that explains the differences in performance. Unfortunately, it is difficult to calculate, e.g., crosslinking density in materials such as the ones produced in the present study due to the fact that information of both the average molecular weight of the monomers (which is a mix of different proteins, where the relative composition of protein might differ) and number of crosslinks is lacking. Simulation tools, which have been used for other plant proteins to understand crosslinking behavior,^60,68^ might be one suitable method forward. Furthermore, LCA and feasibility studies, similar to those carried out for other materials^69,70^ to verify the sustainability and economic aspects of these materials, are required for further understanding of the usefulness of the materials produced here. However, the effect on both swelling and tensile properties from the modification of the protein using either pH 10 + GA or pH 7.5 + Fenton indicates opportunities to use these roads of modifying plant protein to produce a range of different materials including packaging materials (e.g., for food), absorbing materials (e.g., daily-care products), or cushioning materials (health-care products). Of specific interest for these applications is the pH 7.5 + Fenton protein modification as a “green” alternative.

## Supporting Information Available

Table S1: summary of mean values with standard deviation for all measured properties of the unpressed and pressed samples (PDF)
