# Supplementary material for: Green Chemistry to Modify Functional Properties of Crambe Protein Isolate-Based Thermally Formed Films
Source: ACS Omega. 2023 May 31;8(23):20342–51. doi: 10.1021/acsomega.3c00113 (PMC10268266; doi:10.1021/acsomega.3c00113)
Supplement: Supplementary file 1 — ao3c00113_si_001.pdf [file ao3c00113_si_001.pdf]

Green chemistry to modify functional properties of crambe protein isolate based thermally formed films

William R. Newson[a], Antonio J. Capezza[ab], Ramune Kuktaite[a], Mikael S. Hedenqvist[b], Eva Johansson[a]*

[a] Department of Plant Breeding, Box 190, Swedish University of Agricultural Sciences, SE-234 22 Lomma, Sweden

[b] Department of Fibre and Polymer Technology, Royal Institute of Technology, SE-10044 Stockholm, Sweden

*Corresponding Author

*Eva Johansson, Department of Plant Breeding, Box 190, Swedish University of Agricultural Sciences, SE-234 22 Lomma, Sweden

Phone: +46 40 415344.

e-mail: bill.newson@slu.se

Table S1

Sample	Solubility		Mass distribution of soluble protein (arb. units)						Swollen mass/ Protein mass	Tensile properties		
	Unpressed (%)	Pressed (%)	Upressed			Pressed				E modulus (MPa)	Max stress (MPa)	Strain at Maximum Stress (%)
			HMw	MMw	LMw	HMw	MMw	LMw				
**pH4 (no modification)**	33.3(0.4)	10.5(1.2)	0.97(0.12)	47.42(1.73)	51.60(0.84)	1.26(0,60)	12.96(1,52)	17.2(1,17)	1.6(0.2)	57.9(3.1)	2.6(0.3)	11.8(3.0)
**pH10**	37.1(0.3)	4.1(0.3)	1.78(0.34)	62,66(2.08)	46.74(1.57)	0.23(0,39)	2.76(0.73)	9.33(0.92)	3.3(0.3)	38.9(1.7)	3.3(0.2)	116.5(22.1)
**Ph10+GA**	25.7(1.5)	1.6(0.05)	12.78(4.39)	40.80(6,66)	23.54(3.60)	0(0)	0.21(0.13)	4.55(0.18)	2.7(0.1)	36.3(3.2)	4.6(0.3)	157.5(15.6)
**pH10 50C 240min**	39.0(2.5)	5.1(0.2)	4.52(1.25)	70.90(10.38)	41.67(1.41)	0.02(0.02)	3.14(0.20)	12.18(0.27)	4.4(0.2)	41.8(1.2)	3.3(0.2)	151.2(9.1)
**pH10+CA 50C 240min**	39.2(0.7)	5.9(0.2)	5.07(0.57)	72.88(1.04)	39.60(2.03)	0.05(0.01)	4.11(0.16)	13.69(0.25)	4.3(1.0)	48.8(3.0)	3.0(0.2)	103.57(12.3)
**pH7.5 Fenton**	43.3(0.7)	3.7(0.03)	9.53(1.31)	76.42(1.37)	43.89(0.87)	0(0)	0.43(0,03)	10.75(0.13)	2.0(0.03)	39.1(1.3)	3.9(0.2)	157(30.1)

Values in brackets are one standard deviation. HPLC measurements of the mass distribution of soluble protein are absorbance at 210nm, values are a sum of Ex1, Ex2 and Ex3.
